# Annotations

**Published:** 1900-02-17

**Authors:** 


### ANNOTATIONS.

### A Cadet Corps.

WE have learned one thing within the last few months—a thing which we had too much forgotten—that we depend for our national existence on the efficiency of our army. But how is that efficiency to be kept up? Not by regarding the army as the last refuge of the disreputable; though we have of late had wondrous proof that the discipline of the army makes men—and heroes—of those for whom civil life has no use. But we feel now that something should be done to make young men of good quality become soldiers. And the establishment of cadet corps in our great cities would greatly help this. The cadet battalion of the King's Royal Rifle Corps may serve as an instance of the influence of such a corps. A cadet battalion is formed of lads between the ages of 14 and 18, and serves to give healthy evening occupation to boys who are released from the restraints of school, and are free to spend their evenings as they please. Probably they are still sufficiently under the influence of romance to like the idea of soldiering; but no volunteer regiment will take them so young; and, without a cadet corps or something of the kind to give them occupation, they become loafers, picking up all the evil that youth can find in a great city, till they become wretched, lazy, perhaps criminal specimens of humanity. Recently the Government has sanctioned the formation of cadet battalions as part of the volunteer force, and it is to be hoped that those who are interested in volunteering will extend their interest to this form of recruiting for it.

### The R.A.M.C. South Africa Fund.

IT is to be hoped that amid all the evidences of sympathy for, and goodwill towards, the troops in South Africa, which are being shown by every portion of the community at home, the men of the Royal Army Medical Corps will not be forgotten. The territorial system on which our line regiments are raised has led to the establishment in every district of committees for the collection of money and the provision of extra clothing and other comforts for the men connected with each county serving in the county regiments. But the Royal Army Medical Corps has no territorial attachment. It is recruited from the whole country, and thus, being outside local interests, is likely, unless an effort be made, to be left outside the scope of the various funds which almost of necessity are organised on a local basis. Yet there are few men who deserve better of their country than the men of this corps. They are exposed to all the hardships and to a large proportion of the dangers which are faced by the fighting force; and when a battle is over their work, instead of being ended, is in a sense only beginning, for they must then set out on the melancholy task of collecting from the field those wounded whom it has been impossible to bring back from the fighting line while the battle was going on. The Royal Army Medical Corps has worked nobly during this war, and we cannot but think that medical men would do well to bring to the notice of their patients—not the rich ones only, but that vast mass of sick who can give shillings and half-crowns—the claims of the R.A.M.C. Fund, which has been started with the object of supplying extra clothing and comforts for the men. There must be thousands of such patients who would be glad, by subscribing to it, to express their gratitude to and their sympathy with those who are toiling in South Africa to bring comfort and help to the sick and the wounded. By all means let medical men subscribe themselves. Half-a-crown a-piece would go a long way towards such a fund. But medical men can do more than merely putting their hands in their own pockets. They can influence others, and, besides giving and collecting money, they can set to work the willing, although maybe feeble, hands of thousands of convalescents, making flannel shirts and sweaters, knitted helmets, and other comfortable things. This is just the sort of work with which to while away the tedium of recovery, especially when done with such an object. The offices of the fund are at 100, Victoria Street, London, S.W. Money may be sent to the treasurer; and such things as clothing, to the honorary secretary, Miss Maxwell-Müller, at that address.

### Schools and Epidemics.

THE reassertion of the theory of school influence as an active factor in the spread of infectious disease, which has lately been made, makes it desirable to inquire under what circumstances "closure" should be employed. All seems to depend upon whether the assembly of the children in the school in question is the main cause by which the disease is being disseminated. In a country district, for example, with a sparsely scattered population, where children rarely meet except in school, closing the schools at the commencement of an epidemic may effectually check its course, each infected centre working out its own salvation independently, and the one link between the different parts of the district being for the time broken. Again, it may be discovered that in consequence of defective drains or other insanitary arrangements the school is itself actually a cause of disease, or at least that its condition favours the spread of infection among those who attend its classes, and in such a case the school should certainly be closed while the necessary repairs and reconstructions are being carried out. But apart from these two circumstances it must very rarely happen that it is justifiable to close a school, and thus break into the educational progress of the scholars, merely because a large number of them happen to be attacked by an epidemic, for when an epidemic has obtained complete hold in a populous district the school is but one of a dozen ways in which infection is being spread, and it may be far better to keep the school open and thus insure the continuance of that regularity and orderliness of life which attendance at school involves than by closure to throw all the children to play together in the streets or to huddle together in their homes. It must always be remembered that the part played by schools in spreading infection is proportionately greater in the country than in towns, since in the country it is practically in the schools alone that children from different homes come in contact with each other, while in towns the schools are only one out of many centres from which infection may radiate.

